# Combinatorial therapy with an IL-15 superagonist (ALT-803) and anti-PD-L1 mAb augment T cell mediated anti-tumor immunity in mice

**DOI:** 10.1186/2051-1426-2-S3-P234

**Published:** 2014-11-06

**Authors:** Christopher B Johnson, Brian Riesenberg, Dan Neitzke, Emily K Jeng, Warren D Marcus, David Cole, Hing C Wong, Mark P Rubinstein

**Affiliations:** 1 Medical University of South Carolina, Charlestown, SC, USA; 2 Altor BioScience Corporation, Miramar, FL, USA

## 

The adoptive transfer of tumor-reactive T cells has shown great promise in treating patients with metastatic cancer. However, effective T cell responses are limited by the availability of T cell growth factors such as IL-2 and tumor-induced suppressive pathways. As tumor-induced suppression may hamper cytokine responsiveness, we hypothesized that combinatorial therapy providing exogenous cytokine with blockade of inhibitory pathways would lead to synergistic anti-tumor responses. We evaluated this hypothesis by treating mice with palpable B16 melanoma tumors with lymphodepletion and transfer of activated, tumor-reactive CD8^+ ^T cells (pmel-1 TCR transgenic). The persistence of the adoptively transferred tumor-reactive CD8^+ ^T cells was dramatically augmented in the recipient mice with injections of an IL-15 superagonist (ALT-803) which, compared with IL-2, has greater biological activity and does not expand T regulatory cells. The ALT-803-treated mice also survived significantly longer than the untreated mice. B16 melanoma tumor cells were found to express PD-L1 and activated CD8^+ ^T cells have PD-1 on their surface. Thus, we also gave mice anti-PD-L1 mAb treatment to block this PD-1/PD-L-1 inhibitory pathway. Our preliminary data suggest that combinatorial therapy with anti-PD-L1 mAb led to synergistic improvement in anti-tumor efficacy. We are now determining the optimal timing and dosing of ALT-803 and anti-PD-L1 mAb therapy to confirm these results. Currently, ALT-803 is in clinical trials for treating patients with various solid and hematologic tumors. Our findings suggest combinatorial therapy relieving T cell dysfunction using checkpoint inhibitors and providing ALT-803 cytokine therapy may lead to substantially improved outcomes over currently available therapies for patients with metastatic cancer.

